# Supplementary material for: Antibody Responses after SARS-CoV-2 Vaccination in Patients with Liver Diseases
Source: Viruses. 2022 Jan 21;14(2):207. doi: 10.3390/v14020207 (PMC8876976; doi:10.3390/v14020207)
Supplement: Supplementary file 1 [file viruses-14-00207-s001.zip › viruses-1548490-supplementary.pdf]

Supplementary material

(a)

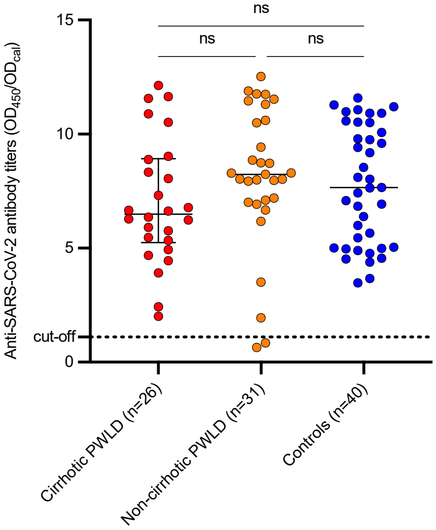

(b)

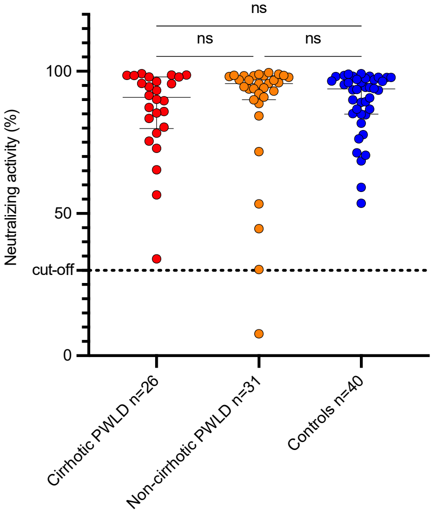

Supplementary Figure S1. Humoral immune responses one month after the second vaccine dose in cirrhotic PWLD, non-cirrhotic PWLD and controls after exlusion of cases receiving immunosuppressive treatment.

Abbreviations: *ns: non-statistically significant; SARS-Cov-2: severe acute respiratory syndrome coronavirus 2; OD: Optical density; PWLD: Patients with liver diseases*
